# On the behaviour of lung tissue under tension and compression

**DOI:** 10.1038/srep36642

**Published:** 2016-11-07

**Authors:** Pinelopi Andrikakou, Karthik Vickraman, Hari Arora

**Affiliations:** 1 Imperial College London, Department of Bioengineering, London, SW7 2AZ, UK; 2 Birla Institute of Technology and Science, Department of Mechanical Engineering, Pilani, 333031, India

## Abstract

Lung injuries are common among those who suffer an impact or trauma. The relative severity of injuries up to physical tearing of tissue have been documented in clinical studies. However, the specific details of energy required to cause visible damage to the lung parenchyma are lacking. Furthermore, the limitations of lung tissue under simple mechanical loading are also not well documented. This study aimed to collect mechanical test data from freshly excised lung, obtained from both Sprague-Dawley rats and New Zealand White rabbits. Compression and tension tests were conducted at three different strain rates: 0.25, 2.5 and 25 min^−1^. This study aimed to characterise the quasi-static behaviour of the bulk tissue prior to extending to higher rates. A nonlinear viscoelastic analytical model was applied to the data to describe their behaviour. Results exhibited asymmetry in terms of differences between tension and compression. The rabbit tissue also appeared to exhibit stronger viscous behaviour than the rat tissue. As a narrow strain rate band is explored here, no conclusions are being drawn currently regarding the rate sensitivity of rat tissue. However, this study does highlight both the clear differences between the two tissue types and the important role that composition and microstructure can play in mechanical response.

## Introduction

The lung parenchyma is one of the most delicate structures within the human body, providing an optimised interface for efficient gas exchange^1^. Its delicate architecture, with its sequential branching down to micro-scale functional units (alveoli), makes it a susceptible organ to trauma. During respiration, the global deformation behaviour of lung tissue is relatively uniform. However, the internal strains that are exhibited by the lung during respiration are locally nonuniform, due to said branching network. This gives rise to local tethering strains in the parenchyma. Furthermore, during any external mechanical loading, resulting from trauma for instance, the response of this structure would also be nonuniform. The reasons for this extend beyond the macro-scale architecture and lie within the micro-scale architecture, the lung constituents, the relative pre-strain within the tissue and the rate or strain-rate differentials in play during the event. This study aims to explore the strain rate sensitivity of lung tissue in the quasi-static regime under tension and compression, to characterise the bulk deformation behaviour. It will also explore the strain-rate behaviour across two species: rat and rabbit tissues.

### Microstructure and Constituents

The alveoli (functional sub-units within the lung) comprise of a monolayer of type I and type II pneumocytes, a thin layer of extracellular matrix (ECM), and the interstitium or endothelial layer of a blood vessel, the type of which depends on the location. The parenchymal ECM is mainly composed not only of mechanically dominant type I collagen, but also type III collagen^2^. Other major constituents of pulmonary ECM are elastin and proteoglycans. Elastin plays an important role in the mechanical function of lung tissue. It has been shown that the macroscopic elastic and viscoelastic properties of alveolar tissue are dominated by both the collagen and elastin fibres^3^,^4^. Elastin has also been proven to be the most important factor in determining recoil for small lung volumes^5^. As such, both collagen and elastin are of significant interest, due to their complementary roles in the biomechanical behaviour of healthy lung tissue. Their relative proportions in the two species, rat and rabbit, explored here may control or affect their mechanical response.

### Lung Composition on Mechanical Performance and Relative Importance

It has been shown that, in human lung samples, the tissue densities of collagen and elastin fibres within 20 *μ*m of an alveolar duct are 18% and 16% respectively^6^. In later studies, quantities were reported to be: 5% and 13% in rats, 4% and 10% in rabbits; and 15% and 29% in humans for their relative elastin and collagen content^7^. *In-situ*, or in its true physiological state, lungs are stressed, as they are a pressure-supported structures, without a defined stress free state as most other organs of the human body^8^. The mechanical behaviour of lungs depends on the level of pre-stress provided by the transpulmonary pressure, as well as the behaviour of its constituent elements^9^. Therefore in mechanical experiments, species-specific behaviour can be expected due to differences in composition. The total amount of collagen fibres in lung parenchyma strips has been correlated with the resistance and elasticity of the tissue, whereas the content of the elastin fibres has been associated with the elastance of the lungs^10^. An important factor affecting the mechanical behaviour is not only the absolute volume of fibres, but also the organization and/or interaction among these fibres^3^,^11^,^12^.

Experimental studies of lung tissue from various species indicate significant variations in composition regarding the proportion of collagen, elastin and other lung tissue constituents. The increase in the thickness of the alveolar interstitium in alveolar duct walls directly depends on the size of the alveoli and their radius of curvature. This increase in the thickness of the interstitium derives from relative increases in both collagen and elastin-rich fibre systems that carry the forces of lung distension in alveoli and alveolar ducts. It was concluded that the mechanical constraints imposed by differences in the radius of curvature of alveoli are responsible for the differences in interstitial and connective tissue fibre thicknesses among the different species^7^. Generally, the fractional content of collagen and elastin fibres is greater in species with larger alveoli, whereas the relative amounts of collagen and elastin fibres are similar. Humans have significantly higher collagen and elastin proportions as compared to other commonly studied laboratory species (rabbits, mice, etc.) as outlined earlier. Mercer *et al*.^7^ did not find significant difference in elastic constants. Furthermore, other components of the alveolar septa such as the epithelium and endothelium do not show a correlation between the alveolar size and interstitial thickness. This means that connective tissue fibres, which are present in the interstitial layer, are the stress-bearing elements while endothelial/epithelial cell layers do not play a significant role in bearing the mechanical stress. Such stress measurements and quantification are beyond the scope of this study.

*Anthropomorphic test devices (ATDs)* are physical human surrogates that have been designed for the specific purpose of representing the human during crash test experiments. The ATDs or dummies are designed to be biofidelic. The prediction of the injury depends on measurements taken from testing the instrumented ATDs during the test^13^,^14^, however, if the mechanical response of these surrogates is inaccurate, then the injury prediction will also be compromised. Therefore, it is of great importance to collect physiologically representative mechanical data, which is the primary aim of this study. This can aid the development of realistically deforming surrogates in the future.

## Results

### Tensile behaviour

Tensile tests were carried out until fracture. However, as different samples fractured at different strains, the properties were studied only up to 60% compression, a point well before the fracture point of all samples. An average curve was plotted for each strain rate tested. Biological tissue exhibits far greater variability than conventional materials but significance values were provided at strains of 15%, 30% and 45% to highlight where significant differences were found between strain rates in each test. A one-way ANOVA with Tukey post-hoc testing was conducted between groups.

**Figure 1:**
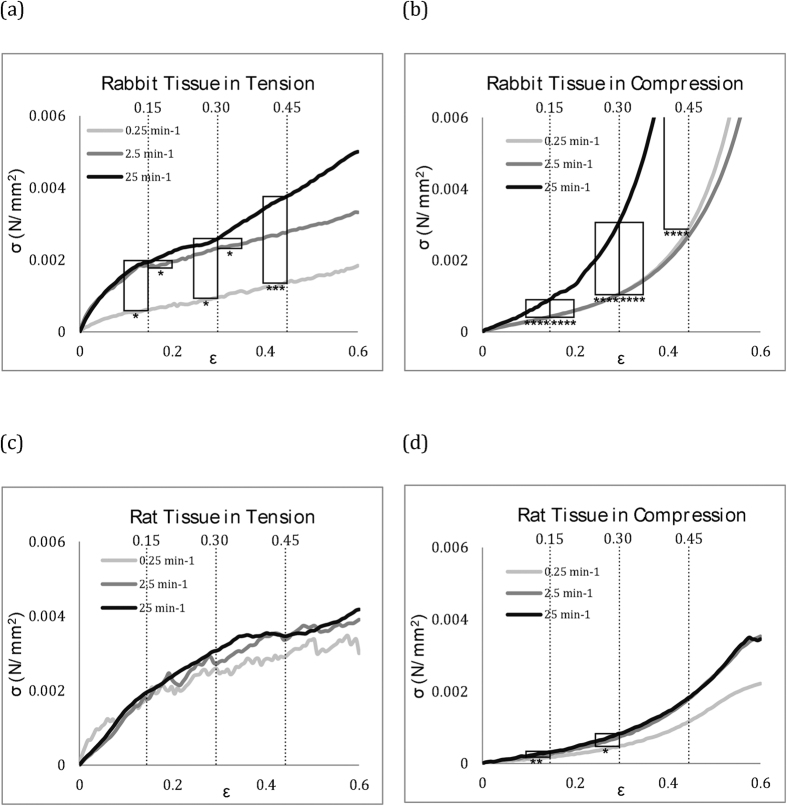
Mechanical test data for rabbit tissue under (**a**) tension and (**b**) compression; and rat tissue under (**c**) tension and (**d**) compression. Average data is presented with significance evaluated between strain rates: *P < 0.05, **P < 0.01, ***P < 0.005 and ****P < 0.0001.

Rat tissue showed very similar qualitative mechanical properties to rabbit tissue under tension (see Fig. 1 for a summary of all the data collected). The strain rate sensitivity is plotted for each tissue comparing across the three different strain rates (0.25, 2.5 and 25 min^−1^). As shown in Fig. 1, higher strain rates correspond to higher values of stresses overall for rabbit lung tissue (with ***P < 0.005 or *P < 0.05). On the contrary, the stress-strain curves representing the rat tissue data do not show significant differences across the strain rates explored in this study. Both sets of tissue are clearly nonlinear in behaviour, but the viscous behaviour of the rat tissue is less apparent than that of the rabbit.

### Compressive behaviour

Compression tests were conducted for both rabbit and rat lung tissue samples using the same strain rates (0.25, 2.5 and 25 min^−1^) and all samples were tested until 60% compressive strain (see Fig. 1). During preliminary experiments, beyond these strains, it became harder to visually monitor and confirm the test validity. However, in terms of useful information for model development, this is still a large strain range, perhaps beyond the upper bounds of requirements.

The lung tissue samples were prepared in the same manner as the tensile specimens with the averaged sample data shown in Fig. 1. In compression, rabbit lung tissue also showed a stiffer initial behaviour compared to rat lung tissue with statistically significant results between the highest rate and the lower rates at ****P < 0.0001; and attained considerably higher stresses, as the highest value for rabbit was approximately 0.03 N/mm^2^ compared to 0.0055 N/mm^2^ in rat.

**Figure 2:**
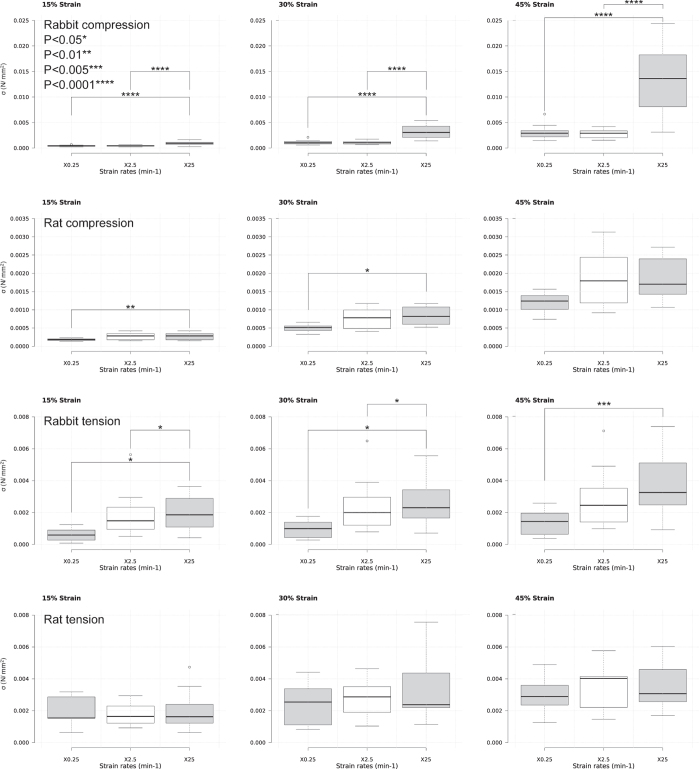
Box plots of raw data with the one way ANOVA with Tukey post-hoc testing analysis at strain intervals of 15%, 30% and 45% for all experimental data. Raw data for all tests are presented with significance evaluated between strain rates: *P < 0.05, **P < 0.01, ***P < 0.005 and ****P < 0.0001.

Comparing between species, rabbit lung tissue data showed once again a stronger rate sensitivity than rat lung tissue data. The rat tissue data seems to imply it is relatively rate insensitive, within this strain rate range, although the highest rate and the lowest rate were varying with significance of *P < 0.05 to **P < 0.01. This needs to be explored further in terms of strain rate range beyond this study to confirm such a characteristic. A summary of the statistical analysis conducted on all raw data is given with box plots in Fig. 2.

### Effect of location of test specimen excision from the global lung

**Figure 3:**
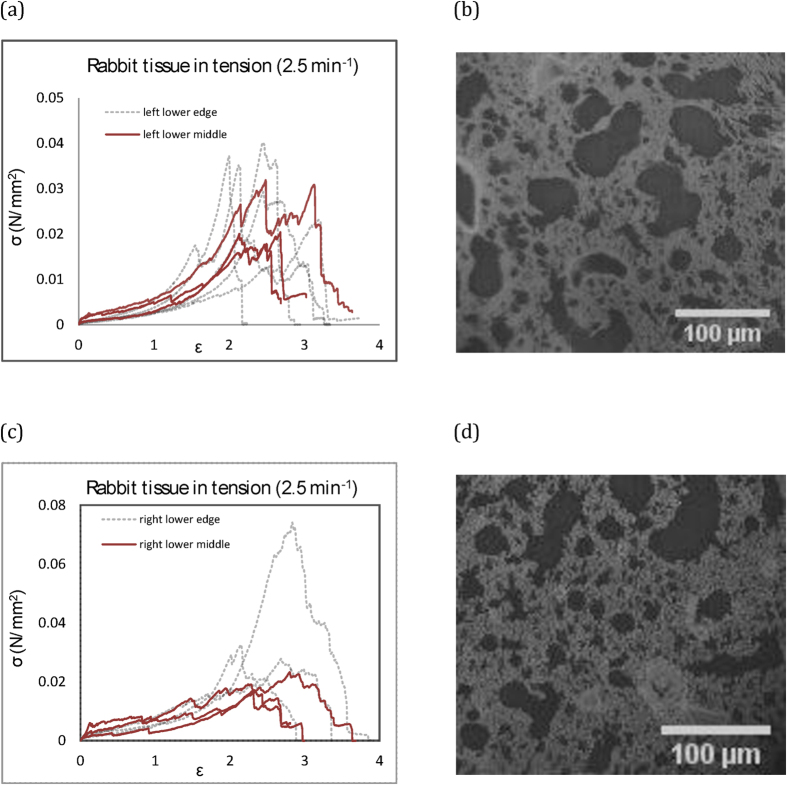
Tensile loading of rabbit lung tissue from different regions of the lung: (**a**,**c**) individual raw data plots; (**b**,**d**) sample histology.

**Figure 4:**
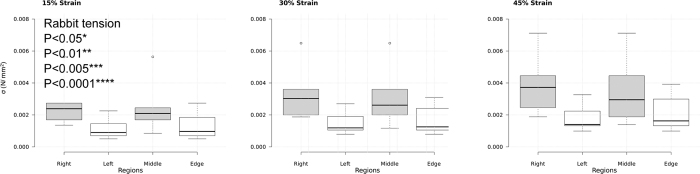
Box plots of raw data with the one way ANOVA with Tukey post-hoc testing analysis at strain intervals of 15%, 30% and 45% for the different regions of the rabbit lung in tension. No significance was found between regions.

The raw data of the tensile rabbit tests are re-analysed and plotted with reference to the biopsy location for the rabbit tissue for one of the rates. It seems that the closer the biopsy to the edge of the global lung sample, the higher the perceived variability of mechanical behaviour (see Fig. 3). The other extreme, towards the primary bronchus, was omitted from this study. This is due to the extreme macro-scale structural heterogeneity near the larger conducting airways, which would dominate the mechanical response, as opposed to a near averaged bulk material experiment. Here, it seems towards the outer edge that variation in response can increase, due to the development of the boundaries (deviating from the developed bulk structure architecture). However, there are no significant differences computed from this set of data below 60%, where our analysis has focused, away from the failure strains. This data was included to confirm that the approach, in terms of sample preparation, was not affected by size of parent lung specimen and location of biopsy. Box plots are also provided for all of the different regions, highlighting no significant differences (see Fig. 4). The analysis was extended to analyse the larger strains up to failure and this showed no significance either.

Sample images from the rabbit tissue are shown in Fig. 3. The images clearly show the highly porous nature of the lungs, however the pore size distribution is visibly not uniform across the section of lung tissue. The microstructure can differ significantly within a single sample from slice to slice by comparing Fig. 3(b,d), as well as from sample to sample. This variation in architecture, partly due to collapse of lung during the preparation methods, but also naturally occurring variability from sample to sample, contributes towards the biological variation observed overall in all experiments presented.

## Discussion

According to Suki and Bates^15^, lung parenchymal tissue shows both elastic and dissipative mechanical properties, as well as highly non-linear characteristics. Compression and tension tests at different strain rates (0.25, 2.5 and 25 min^−1^) demonstrated the nonlinear elastic and nonlinear viscoelastic mechanical behaviour of rat and rabbit lung tissue. To explore this further and to quantify such viscous phenomena analytical models traditionally used in biomaterial science were applied to our tissue data to characterise each tissue.

**Figure 5:**
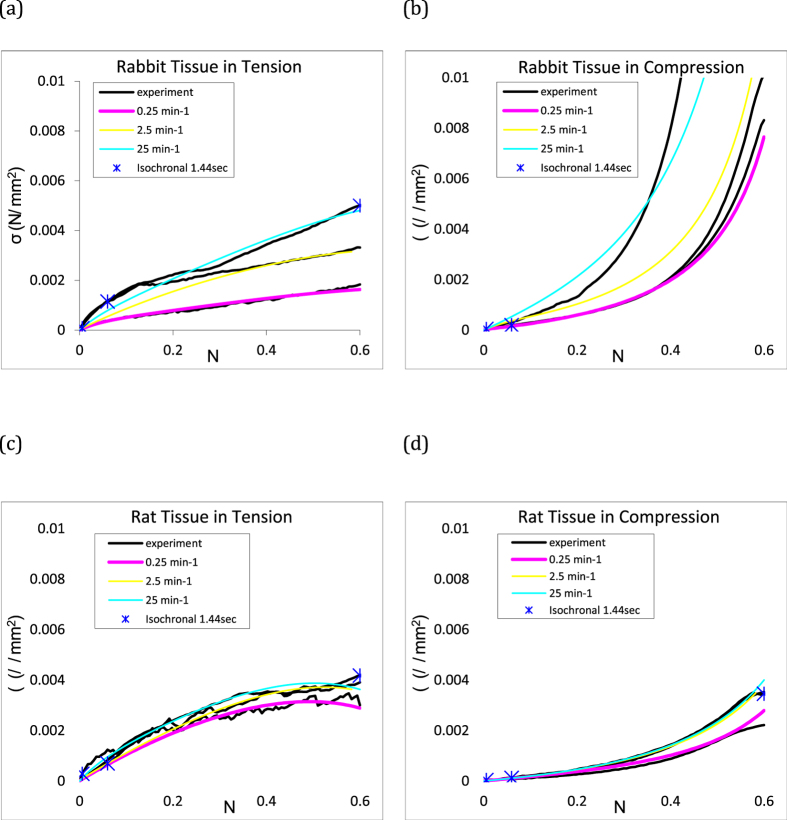
The average tensile and compressive loading data for all strain rates (0.25, 2.5 and 25 min^−1^) for the rabbit tissue (top) and rat tissue (bottom) data, including the fit to the nonlinear viscoelastic model using the van der Waals hyperelastic potential.

The tissue exhibits a non-linear σ − *ε* response, as illustrated by the example isochronal plot at a time of 1.44 s in Fig. 5. However, it was possible to accurately characterise the tissue behaviour with a hyper viscoelastic material law which is shown in Fig. 5.

Assuming a separable time- and strain-dependent material behaviour^16^,^17^, the relaxation stress under a step strain loading history is written as:


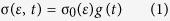


where *g*(t) and σ_0_(*ε*) are functions of time and strain respectively. The chosen form of the time function is the Prony series^16^:


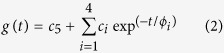


where *t* and *ϕ*_*i*_ are time and time constants respectively, and *c*_*i*_ are dimensionless constants related to *c*_5_ through 
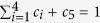
. For an arbitrary strain history, the stress is evaluated via the Leaderman form of the superposition integral^18^:


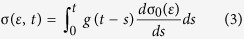


where σ_0_(*ε*) is the instantaneous stress at strain *ε*. The van der Waals model is chosen as the hyperelastic potential^19^. The true stress form for uniaxial tension and uniaxial compression can then be described using 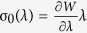, where *W* is the hyperelastic potential and *λ* is the stretch ratio equal to the exponential of the current true strain *ε*. The stress for uniaxial tension, σ_0_(*ε*), can then be derived as:


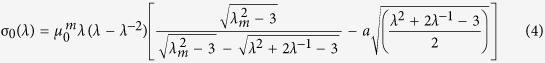


where 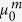 is the instantaneous initial shear modulus, *λ*_*m*_ is the locking stretch constant and *a* is the global interaction parameter^20^.

By substituting Eq. 2 in Eq. 3, and evaluating the integral using finite time increments^21^,^22^, the following form is obtained:


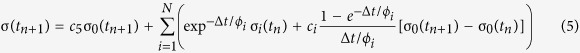


Equation 5 is a function for updating the stress σ(*t*_*n*+1_) where Δ*t* is the time increment defined as Δ*t* = *t*_*n*+1_−*t*_*n*_ and σ_*i*_(*t*_*n*_) is the stress corresponding to the *i*^*th*^ term of the Prony series at time *t*_*n*_ i.e. 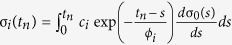. Eq. 5 can be used to calculate the stress at any time, *t*_*n*+1_, provided the stress at the previous time increment, *t*_*n*_, is known. The initial state at *t* = 0 is σ = *ε* = 0, hence the stress at *t* > 0 can be calculated. A detailed derivation is given in ref. 16. The function can be evaluated for various strain histories by minimizing the error between the experimental values of stress and those predicted using Eq. 5. The time constants *ϕ*_*i*_ are fixed. A five term Prony series was sufficient to give a good fit to experiment up to a strain of 0.5 as shown in Fig. 5. All parameter values are summarised in Table 1.

This model, and many other material models commonly used in soft tissue biomechanics, are readily available in commercial finite element packages. Once higher strain rate data is collected, such models can further build in complexity, incorporating a broader strain-rate range, and enable more detailed thoracic deformations to be evaluated accurately *in-silico*.

It has been shown by Booth *et al*.^23^, that native normal human lung tissue has stiffness of ≈2 kPa. This study showed comparable values for rabbit and rat tissues, however it also highlighted the nonlinear nature of the tissue as well as the effect of strain rates on these properties. It is important to report such characteristics in order to fully describe the mechanics behind such tissue behaviour. Regarding the rabbit tissue, it showed higher instantaneous stiffness at higher rates. An example analytical model (van der Waals hyperelastic function in conjunction with a five term Prony series) were applied to this data and reported for the benefit of numerical modelling methods.

Rabbit and rat lung tissue showed different mechanical behaviour, and both behaved differently in compression compared to tension. The maximum stress value for rabbit under compression is approximately 0.03 N/mm^2^ compared to 0.0055 N/mm^2^ in rat. Although similar sized samples were extracted from both animals, rat lung tissue showed less variation in the stress-strain curves comparing to rabbit lung tissue. This is likely to be due to the jump in length scales from rat to rabbit, leaving the rat samples relatively rate insensitive at the strain rates examined in this study.

## Methods

### Materials

**Figure 6:**
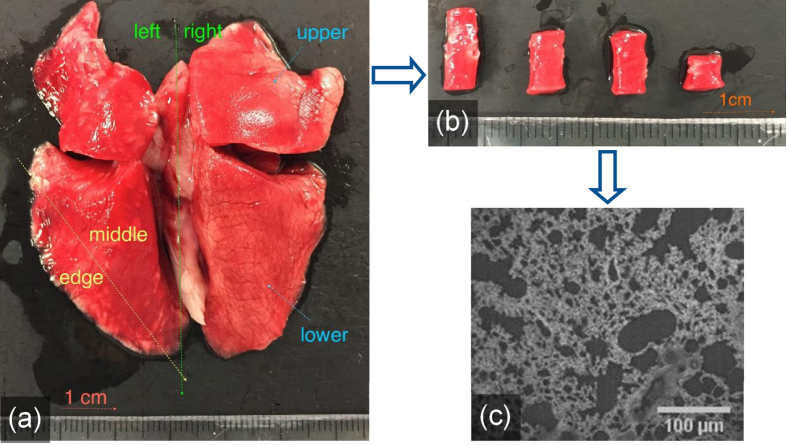
Rabbit lung tissue: (**a**) the area of biopsy locations highlighted; (**b**) test samples of varying quality for assessment; and (**c**) a micrograph of the excised tissue.

All lung tissue was excised from fresh cadaveric rabbits (see Fig. 6) and rats and tested on the same day. Rabbits were male New Zealand White rabbits (HSDIF strain, Specific Pathogen Free, Harlan UK) weighing between 2.15 to 3.35 kg and they were 3 months old. All rats were also male, Sprague Dawley, weighing ~0.4 kg, and their age was 3.5 months. No live animals or human subjects were tested in this study. Cadaveric tissue was obtained from animals after procedures approved by the Imperial College London ethical review process and strictly conformed to the Animals (Scientific Procedures) Act 1986 UK Home Office guidelines, which also fulfil the US NIH Guide for the Care and Use of Laboratory Animals. All lungs were collected at Imperial College London, before being stored in phosphate buffer saline (PBS) at 4 °C, until usage. The storage in PBS and the cutting procedure is assumed to have minimal effect on the mechanical properties of the tissue. Mechanical testing was undertaken within 6 hours of lung extraction. Recent publications from the group have highlighted the importance of preparation and storage techniques on mechanical properties of soft tissues^24^. Elsewhere rat lung samples, for example, have been prepared using specialised techniques to live for up to 48 hours of the animal’s death^25^,^26^. While others have shown that the mechanical properties of these samples are not affected within 72 hours of death^27^. Edge effects and excessively large sections of conducting airway (collagen rich tubing) in samples were eliminated from the study either at the point of cutting or after test completion, where these structures became visibly disruptive to uniform tissue deformation.

Punches of 8 mm diameter were used to cut perpendicular to the frontal plane of the lungs. Height of the specimens varied between 3.6–10.0 mm. The relative location of each test sample (from within a single set of lungs) was recorded, as shown in Fig. 6. This was to ensure variability in mechanical behaviour could be interrogated in terms of expected homogeneity of the microstructure within the global lung sample. Sample preparation quality was also assessed, in terms of cut and finish, prior to testing.

The lung parenchyma is expected to be more uniform in microstructure towards its periphery, where it is primarily made up of alveolar sacs, rather than the primary branching towards the centre. Spatial variations in microstructure and local mechanics are important to this study. The lungs are a very soft organ and their compressibility inevitably lead to variability in sample geometries. Although the punch is of 8 mm diameter, the tissue samples extracted showed a variation in their diameter (even within the length of a single sample). Therefore multiple measurements of the diameters were taken along each specimen length before each experiment. Samples with largely varying cross-sections were removed from the test program. Once prepared, exposure time of the lung tissue outside of PBS was kept to a minimum to limit tissue dehydration effects.

## Experimental

### Mechanical test arrangement

Both the compressive and tensile experiments were carried out using a 10N Load Cell on a Universal Testing Machine (Zwick Roell). For the tensile tests aluminium platens were used as sample mounts and the samples were fixed in place using Ethyl Cyanoacrylate. Samples were recovered for histological analysis and the platens polished between uses. Edge effects due to the bonded interface are assumed to be negligible at small strains. This is due to the foam-like nature of the lung, meaning a Poisson’s ratio tending towards 0 would result in no (or minimal) contraction during axial loading. At large strains (>0.5) and particularly at failure, this breaks down, however the initial behaviour can be assumed to be uniaxial.

For compressive tests, samples were directly placed on paraffin oil coated PTFE plates to minimise friction and negate possible barrelling effects. One of the limitations of these mechanical tests is the fact that the volume of air ‘trapped’ in each sample was not controlled. This was highlighted previously as a key factor in lung tissue behaviour. Future work will address the shortcomings of this experiment in terms of physiological robustness. This study aims to provide data on the bulk properties of the tissue itself, rather than determining properties present at an instant *in-vivo*. Samples were also collected and recovered in order to provide a control for sample quality. Evaluation of sample quality was conducted later in the study (see Histology and optical imaging). Deformation, time and force data were recorded for each sample and then analysed to obtain stress-strain curves for all samples. For all experiments, the strain rates used were 0.25, 2.5 and 25 min^−1^.

A total of 161 tests were conducted with 29 discarded due to visibly asymmetric deformation either due to their bond or interaction with the grips or the presence of a large conducting airway. A full summary of the dataset collected and taken forward for analysis is given in Table 2.

### Histology and optical imaging

Samples were recovered after each experiment and prepared for paraffin wax embedding for histological analysis. The protocol used involved dehydration phases using ethanol and xylene. Finally, the tissues were infused with a 0.5% Sudan IV (red) dye in xylene sourced from Sigma-Aldrich^28^. The wax was stained with a 1% Sudan II (blue) dye and introduced under vacuum to fill the airways and vessels within the lung tissue. These were then cast in conventional histology cassette blocks ready for sectioning and imaging.

An automated sectioning and imaging suite, called the Histocutter, has been developed in-house at the Department of Bioengineering, Imperial College London. This robotic device expertly constructs high-throughput, high resolution, 3D histological imaging, capturing and aligning thousands of multi-spectral high quality images and making them ready for quantitative analysis and 3D visualisation. This functions much like conventional manual process of sectioning and imaging using optical light microscopy but is automated within the platform using a calibrated control system. This was used to image a single sample from each batch of lung samples tested, and thus to ensure comparable architectural properties remained for each batch of samples.

## Additional Information

**How to cite this article**: Andrikakou, P. *et al*. On the behaviour of lung tissue under tension and compression. *Sci. Rep.* **6**, 36642; doi: 10.1038/srep36642 (2016).

**Publisher’s note:** Springer Nature remains neutral with regard to jurisdictional claims in published maps and institutional affiliations.

## Figures and Tables

**Table 1:** Experimentally calibrated parameters for the van der Waals based hyper-viscoelastic model are shown for each tissue and loading mode, where the instantaneous shear modulus is 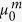 , a locking stretch, *λ*_*m*_ and global interaction parameter, *a*.

Rabbit tension							
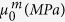	*λ*	*a*	*c*_1_	*c*_2_	*c*_3_	*c*_4_	*c*_5_
0.006	7500	0.50	0.47	0.00	0.37	0.00	0.16
Rabbit compression							
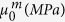	*λ*	*a*	*c*_1_	*c*_2_	*c*_3_	*c*_4_	*c*_5_
0.003	2.4	0.0	0.00	0.78	0.00	0.00	0.22
Rat tension							
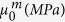	*λ*	*a*	*c*_1_	*c*_2_	*c*_3_	*c*_4_	*c*_5_
0.007	1400	0.77	0.46	0.00	0.00	0.13	0.41
Rat compression							
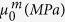	*λ*	*a*	*c*_1_	*c*_2_	a*c*_3_	*c*_4_	*c*_5_
0.0006	2.6	0.0	0.00	0.00	0.00	0.99	0.01
			*ϕ*_1_ (s)	*ϕ*_2_ (s)	*ϕ*_3_ (s)	*ϕ*_4_ (s)	*ϕ*_5_
			0.1	1	10	100	∞

**Table 2:** Summary of all tests conducted.

Tissue Species	Mode	Rate (min^−1^)	N
NZW	Tension	0.25	7
NZW	Tension	2.5	17
NZW	Tension	25	15
SD	Tension	0.25	5
SD	Tension	2.5	7
SD	Tension	25	10
NZW	Compression	0.25	15
NZW	Compression	2.5	17
NZW	Compression	25	12
SD	Compression	0.25	7
SD	Compression	2.5	10
SD	Compression	25	10
